# Surgical outcomes of endoscopic enucleation of the prostate in community aging males with or without preoperative urinary retention

**DOI:** 10.1007/s11255-024-04007-7

**Published:** 2024-04-02

**Authors:** Tung-Shiun Hsu, Shu-Chuan Weng, Yu-Hsiang Lin, Chien-Lun Chen, Shu-Han Tsao, Han-Yu Tsai, Horng-Heng Juang, Phei-Lang Chang, Chen-Pang Hou

**Affiliations:** 1 Department of Urology, Chang Gung Memorial Hospital at Linkou, Taoyuan, 333 Taiwan; 2 Department of Health and Management, Yuanpei University of Medical Technology, Hsinchu, 330 Taiwan; 3 Bachelor Degree Program of Senior Health and Management, Yuanpei University of Medical Technology, Hsinchu, 330 Taiwan; 4 School of Medicine, Chang Gung University, Taoyuan, 333 Taiwan

**Keywords:** Prostate, Enucleation, Benign prostate hyperplasia, Thulium laser, TURP, Urinary retention

## Abstract

**Objectives:**

This study aims to investigate the surgical outcomes of endoscopic enucleation of the prostate in older males with or without preoperative urinary retention (UR).

**Material and methods:**

We conducted a study on selected patients with symptomatic benign prostatic hyperplasia (BPH) who underwent either thulium:YAG laser (vela XL) prostate enucleation (ThuLEP) or bipolar plasma enucleation of the prostate (B-TUEP) at the geriatric urology department of our institution. The studied patients were categorized into two groups, namely the UR group and the non-UR group, on the basis of whether they experienced UR in the 1 month preceding their surgery. Their clinical outcomes following prostate endoscopic surgery were evaluated and analyzed.

**Results:**

Our results revealed comparable outcomes for operation time, length of hospital stay, percentage of tissue removed, re-catheterization rate, and urinary tract infection rate within the 1 month between the B-TUEP and ThuLEP surgery groups, regardless of UR history. However, the non-UR B-TUEP group experienced more blood loss relative to the non-UR ThuLEP group (*P* = .004). Notably, patients with UR exhibited significantly greater changes in IPSS total, IPSS voiding, and prostate-specific antigen values relative to those without UR.

**Conclusions:**

Both ThuLEP and B-TUEP were effective in treating BPH-related bladder outlet obstruction. Our study identified more pronounced changes in IPSS total, IPSS voiding, and prostate-specific antigens within the UR group. Moreover, the rate of postoperative UR in this group was not higher than that observed in the non-UR group. Our study also revealed that the presumed benefits of laser surgery in reducing blood loss were less pronounced for patients with UR.

## Introduction

Benign prostatic hyperplasia (BPH) refers to the histological diagnosis of the proliferation of glandular tissue within the prostate. This condition is prevalent among older men, affecting approximately 50% of men aged 50 to 60 years and > 70% of those aged 80 to 89 years [1]. Urinary retention (UR) is among the most severe symptoms associated with BPH, with a reported cumulative incidence rate of approximately 20% for men aged 50 to 89 years [2]. Urinary retention is characterized by the inability to void, if left unrecognized and untreated, it has the potential to escalate into a serious condition, posing risks such as kidney damage or urosepsis and jeopardizing the patient’s life. [3]. UR management typically involves immediate catheterization combined with an alpha-blocker. However, in a study of patients with UR, the success rate of trials without a catheter was 61.4% and 29.5% during the first and second attempts, respectively [4]. As such, surgical intervention is often required. Transurethral resection of the prostate (TURP) has been the standard surgical intervention for benign prostatic obstruction (BPO) for 40 years [5]. In recent years, laser technology has approached the treatment efficacy of traditional TURP, and transurethral enucleation with bipolar energy has emerged as a viable prostate surgery option with reasonable effectiveness and low risk [6, 7]. Although these two methods are increasingly accepted, few studies have compared their prognoses, particularly for patients with UR. Thus, the present study aims to investigate the surgical outcomes of the two procedures in aging males with or without preoperative UR.

## Materials and methods

### Patient selection and evaluation

This study was a retrospective interpretation of prospectively acquired data of selected patients with symptomatic BPH who underwent either 120-W thulium:YAG laser (vela XL) prostate enucleation (ThuLEP) or bipolar plasma enucleation of the prostate (B-TUEP) at the geriatric urology department of Chang Gung Memorial Hospital in Taiwan. It was conducted between October 2018 and July 2022 after it was approved by the institutional review board of Chang Gung Memorial Hospital (IRB number: 202101983B0). All the patients were operated on by a single skilled surgeon. A TRUS biopsy was performed when there was suspicion of prostate cancer in our patients, indicated by abnormal DRE findings, PSA levels exceeding 4 ng/ml, or the identification of a hypoechoic lesion in the TRUS images. Voiding ability was assessed through uroflowmetry, and the recorded data included voiding volume (VV), peak flow rate (Qmax), uroflow figure, and post-void residual (PVR). International Prostate Symptom Scores (IPSSs) and IPSS Quality of Life (QoL) scores were also recorded. Patients were included in the present study if they had a prostate volume of > 30 cm^3^, an IPSS of > 20, a Qmax of < 15 ml/s, and an Eastern Cooperative Oncology Group performance status score of < 2 [8]. All the included patients had been undergoing medical treatment for BPH for at least 3 months prior to their surgery, and they also met the surgical requirements for BPO [9]. Patients with a history of prostate surgery or active malignant disease were excluded, as were patients with neurogenic bladder or lower urinary tract symptoms unrelated to BPH. Patients were included in the UR group if they were admitted with a urinary catheter or if they were admitted without a urinary catheter but had been catheterized for urinary retention within 1 month preceding admission. All recorded preoperative variables, including PSA, were derived from the most recent examination data conducted before being catheterized.

### Surgical equipment and techniques

Patients who underwent B-TUEP were treated using an Olympus SurgMaster UES-40 bipolar generator and OES-Pro bipolar resectoscope (Olympus Europe, Hamburg, Germany). The surgical technique used in the present study was performed per the procedure outlined by Liu et al. [10]. For all laser enucleation procedures, a 120-W thulium laser (Vela XL, Boston Scientific, Marlborough, MA, USA) that emits a continuous wavelength of 1.94 μm was employed. The blood loss during the enucleation procedure was assessed using a low hemoglobin (Hb) photometer (HemoCue, Ängelholm, Sweden) [11] and calculated by applying the following formula: Hb concentration in irrigant (g/dL) × volume of irrigant [ml])/preoperative blood Hb concentration (g/dL). Prior to the procedure, the patients underwent blood extraction, and their blood Hb concentration was determined. During the procedure, the patient’s Hb concentration was determined by analyzing the collected irrigants. To prevent blood coagulation, 15 000 IU of heparin was added to each 10-L container storing the irrigants collected during surgery [12]. Antibiotics were administered both before and after surgery per the recommended protocol [13].

### Outcome evaluation at follow-up

The patients attended follow-up appointments at 2 weeks, 3 months, and 6 months after they were discharged from the hospital. During these visits, evaluations were conducted to obtain the patients’ IPSS score, QoL score, Qmax, VV, uroflow figure, and PVR.

### Statistical analysis

The present study conducted chi-square tests and independent samples t-tests to examine the differences between the UR group and non-UR group in terms of age, prostate size, treatment duration, and several preoperative values, namely IPSS (IPSS total, IPSS voiding, and IPSS storage), Qmax, VV, PVR, and medication use. Furthermore, repeated measures analysis of variance was performed to identify differences in IPSS, QoL, Qmax, VV, and PSA changes between the UR group and non-UR group before surgery, 2 weeks after surgery, 3 months after surgery, and 6 months after surgery. The significance level for all statistical analyses was set at *P* < 0.05. The statistical software SPSS version 25.0 was used for all data analysis.

## Results

### Baseline characteristics

There were a total 207 patients included in our study (non-UR group: 153; UR group: 54). The baseline characteristics of the patients are presented in Table 1. Relative to the non-UR group participants, the UR group participants were older (UR, 70.41 ± 8.54 years; non-UR, 66.53 ± 8.46 years; *P* = 0.004) and exhibited a higher PSA level (UR, 8.61 ± 8.81; non-UR, 4.88 ± 4.65; *P* = 0.006). The UR group did not differ significantly from the non-UR group in terms of creatinine level or medication duration. Despite experiencing urination difficulties, the UR group participants did not manifest a larger prostate or transition zone. For comorbidities, the UR group did not differ significantly from the non-UR group in the incidence of diabetes, hypertension, coronary heart disease, chronic renal disease, or arrhythmia; however, the group exhibited a higher prevalence of stroke (UR, 11 [20.37%]; non-UR, 6 [3.9%); *P* < 0.001).

**Table 1:** Baseline characteristics of the patients

Parameter (mean ± SD) (*n*, %)	Non-UR *N* = 153	UR *N* = 54	*P* value
Age (years)	66.53 ± 8.46	70.41 ± 8.54	0.004
PSA (μg/l)	4.88 ± 4.65	8.61 ± 8.81	0.006
Cr (mg/dl)	1.00 ± 0.58	1.05 ± 0.41	0.511
Prostate volume (ml)	52.19 ± 19.21	55.35 ± 25.27	0.341
Prostate T zone (ml)	23.55 ± 13.29	26.92 ± 17.22	0.196
Medication duration (months)	26.43 ± 38.60	28.94 ± 45.91	0.697
Thulium laser (*n*, %)	88(57.5%)	29(53.7%)	0.627
Comorbidities (*n*, %)			
DM	29(19.0%)	13(24.1%)	0.421
HTN	73(47.7%)	28(51.9%)	0.601
CAD	12(7.8%)	5(9.3%)	0.745
Arrhythmia	8(5.2%)	6(11.1%)	0.139
Stroke	6(3.9%)	11(20.4%)	< 0.001
CRI	10(6.5%)	3(5.6%)	0.798
IPSS (total)	24.10 ± 4.69	25.24 ± 4.78	0.128
IPSS (voiding)	14.98 ± 3.40	15.87 ± 2.94	0.089
IPSS (storage)	9.12 ± 3.51	9.37 ± 3.39	0.648
IPSS (QoL)	4.57 ± 0.72	4.83 ± 1.11	0.112
Qmax (ml/s)	8.67 ± 3.30	7.06 ± 4.82	0.062
VV (ml)	199.91 ± 107.97	170.49 ± 111.26	0.141
PVR (ml)	85.16 ± 99.44	232.16 ± 250.83	0.001
Duration of medication (month)	26.43 ± 38.60	28.94 ± 45.91	0.697
α-blockers	151(98.7%)	52(96.3%)	0.279
β3 agonist	6(3.9%)	1(1.9%)	0.679
Anti-muscarinic	33(21.6%)	5(9.3%)	0.045
Bethanechol	10(6.5%)	2(3.7%)	0.735
DDAVP	6(3.9%)	2(3.7%)	0.943

*SD:* Standard deviation, *PSA:* prostatic-specific antigen, *Cr:* creatinine, *DM:* diabetes mellitus, *HTN:* hypertension, *CAD:* coronary arterial disease, *CRI:* chronic renal insufficiency, *IPSS:* International Prostate Symptom Score, *QoL:* quality of life, *Qmax:* maximum flow rate, *VV:* voiding volume, *PVR:* post-voiding residual urine, *UR:* urinary retention, *DDAVP:* deamino D-arginine vasopressin

### Preoperative status

The UR group and non-UR group did not differ significantly in their IPSSs (IPSS voiding, IPSS storage, and IPSS total) or in QOL, as shown in Table 1. For urodynamics, no significant differences in Qmax and VV were identified between the two groups, but the UR group exhibited a significantly elevated PVR relative to the non-UR group (UR, 232.16 ± 250.83; non-UR, 85.16 ± 99.44). Regarding medication use for urination difficulties, both groups exhibited no significant differences in the use of alpha-blockers, beta-3 agonists, bethanechol, and DDAVP; however, the non-UR group exhibited a higher prevalence of antimuscarinic use (UR, 5 [9.3%]; non-UR, 33 [21.6%]; *P* = 0.045).

### Intraoperative and perioperative data

Table 2 presents the intraoperative and perioperative data obtained in the present study. The duration of surgery, length of hospital stays, percentage of T zone tissue removed, rate of re-catheterization, and rate of urinary tract infections (UTIs) within 1 month after surgery did not differ significantly between the UR group and the non-UR group, regardless of whether the patients underwent B-TUEP or ThuLEP. No patient required a blood transfusion during their surgery, and urinary incontinence did not occur postoperatively in both groups. However, for blood loss, patients in the non-UR group who underwent B-TUEP experienced more blood loss relative to those who underwent ThuLEP (non-UR B-TUEP, 214.08 ± 285.18 ml; non-UR, ThuLEP, 96.58 ± 143.10 ml; *P* = 0.004). In addition, patients in the UR group who underwent B-TUEP were more likely to return to the emergency room (ER) within 1 month after surgery relative to the non-UR and UR ThuLEP groups (non-UR ThuLEP, 6 [6.8%]; UR B-TUEP, 8 [32%]; UR ThuLEP, 2 [7.9%]; *P* = 0.009).

**Table 2:** Intraoperative and perioperative data

Parameter (mean ± SD) (*n*, %)	Non-UR (65) B-TUEP(1)	Non-UR (88) ThuLEP(2)	UR (25) B-TUEP(3)	UR (29) ThuLEP(4)	*P* value	Post hoc (Tukey)
OP time (minute)	86.17 ± 47.83	80.39 ± 30.25	93.92 ± 52.37	85.00 ± 33.22	0.486	
Estimated blood loss (ml)	214.08 ± 285.18	96.58 ± 143.10	175.93 ± 201.22	112.37 ± 125.62	0.004	(1) > (2)
Hospitalization duration (days)	2.31 ± 0.79	2.10 ± 0.43	2.32 ± 0.69	2.24 ± 0.58	0.160	
Percentage of T zone tissue removed (%)	81.93 ± 41.91	87.71 ± 58.85	81.22 ± 40.05	97.97 ± 43.32	0.508	
Blood transfusion (*n*, %)	non					
Re-catheterization within 1 month (*n*, %)	3(4.6%)	6(6.8%)	2(8%)	0(0%)	0.493	
UTI within 1 month (*n*, %)	24(36.9%)	29(33.0%)	7(29.2%)	6(20.7%)	0.466	
Returned to ER within 1 month (*n*, %)	10(15.4%)	6(6.8%)	8(32.0%)**	2(7.9%)	0.009	(3) > 2 (3) > 4

*SD:* standard deviation, *OP:* operation, *UR:* urinary retention, *B-TUEP:* bipolar plasma transurethral enucleation of the prostate, *ThuLEP:* thulium:YAG laser (vela XL) prostate enucleation, *UTI:* urinary tract infection, *AGE:* acute gastroenteritis

### Postoperative follow-ups

**Fig. 1:**
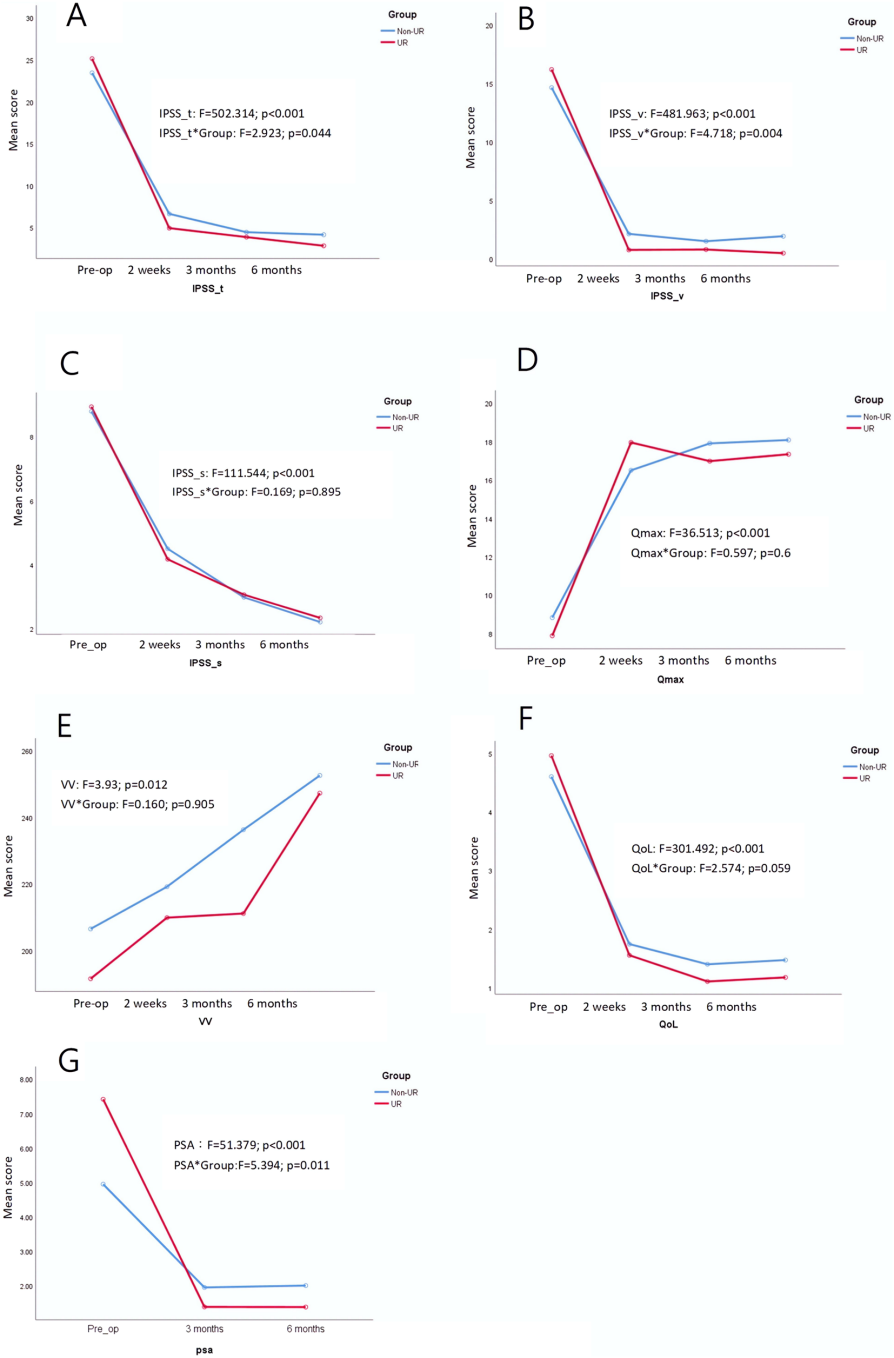
**A** Alterations in IPSS_t following the surgical procedure. The analysis found an interaction effect between the UR status and the values of IPSS_t at different time points, indicating that the changes in IPSS_t were significantly greater in the UR group than in the non-UR group. **B** the alterations in IPSS_v following the surgical procedure. The analysis found an interaction effect between the UR status and the values of IPSS_v at different time points, indicating that the changes in IPSS_v were significantly greater in the UR group than in the non-UR group. **C** The alterations in IPSS_s following the surgical procedure. **D** Significant improvements were observed in Qmax following the surgical procedure. **E** Significant improvements were observed in QoL VV following the surgical procedure. **F** Significant improvements were observed in QoL scores following the surgical procedure. **G** The changes in PSA level following the surgical procedure. The analysis found an interaction effect between the UR status and the values of PSA at different time points, indicating that the changes in PSA were significantly greater in the UR group than in the non-UR group

Figure 1A–C present the changes in IPSSs following surgery, highlighting the considerable improvements that the patients experienced with respect to their IPSS total, IPSS voiding, and IPSS_s results after surgery. Similarly, the patients achieved significant improvements with respect to their Qmax (Fig. 1D), VV (Fig. 1E), IPSS-QoL (Fig. 1F), and PSA (Fig. 1G). Furthermore, an analysis revealed an interaction effect between UR status and the parameters of IPSS total, IPSS voiding, and PSA at multiple time points, indicating that the changes in IPSS total, IPSS voiding, and PSA values were significantly greater in the UR group than in the non-UR group.

## Discussion

TUEP is comparable to TURP in terms of its efficacy, functional outcomes, and risk of complications in treating bladder outlet obstruction (BOO) [14–18]. Fusco and colleagues suggested that bladder outlet obstruction (BOO), primarily arising from benign prostate enlargement, results in gradual remodeling of the bladder [19]. The mechanical strain caused by BOO initiates hypertrophy and angiogenesis in the bladder wall [20]. BOO has the potential to induce prolonged tissue hypoxia, leading to various remodeling changes such as smooth muscle loss, neuronal damage, and the deposition of extracellular matrix [20]. Additionally, a separate study demonstrated that persistent BOO could lead to urinary retention. The researchers observed significantly higher levels of intrafascicular collagen in individuals with a history of acute urinary retention (AUR) compared to those without such a history [21]. Therefore, an effective treatment to prevent bladder tissue remodeling is very important. Notably, studies have investigated the surgical effect of prostate endoscopic surgery in treating AUR [22–25]. However, these studies have primarily analyzed conventional techniques, such as TURP. To the best of our knowledge, our research is the first study focused on the effectiveness of new techniques (e.g., B-TUEP and ThuLEP) for patients with AUR and the differences in clinical outcomes between patients with AUR and without AUR.

In our study, the AUR group was significantly older (*P* = 0.004) relative to the non-AUR group. Consequently, older patients with prostatic hypertrophy may experience AUR as the most severe form of obstructive symptoms [26], indicating that older age is linked with an increased risk of AUR in community-dwelling men [26]. In our study, the PSA level in the UR group was also notably higher than that in the comparison group. PSA is not a cancer-specific serum marker, and various physiologic and benign pathologic processes can affect serum PSA concentration, including prostatitis, UR, ejaculation, and external compression [27].

In our study, the UR group exhibited more significant reductions in IPSS and PSA levels but not in Qmax relative to the non-UR group. A previous meta-analysis of 25 studies indicated that patients with UR achieved a more significant improvement in IPSS at the 3-month follow-up assessment but not at the 6-month and 12-month follow-up assessments [28]. Compared to these literature findings, our findings differ slightly. This difference may be attributed to the fact that the study above examined the patients who underwent different kinds of endoscopic procedures. In contrast, our study specifically focused on analyzing patients who underwent prostate enucleation. Several studies have reported that individuals diagnosed with BPH and UR exhibit a greater incidence of short-term postoperative complications compared with patients with only LUTS [24, 28]. A retrospective study revealed that ThuLEP led to less blood loss relative to B-TUEP [29]. In our study, no statistically significant difference in postoperative complications, such as UTI, was identified between the AUR group and non-AUR group. The blood loss reduction achieved through ThuLEP was observed only in the non-UR group. In a retrospective review of 213 patients who underwent holmium laser enucleation of the prostate, both the LUTS and UR groups exhibited a 3% UR rate and required temporary re-catheterization (*P* = 1) [25]. Likewise, our study did not identify any significant difference in postoperative re-catheterization rates between the UR group and the non-UR group within 1 month after surgery. This finding aligns with that of Johnson et al. [25].

The present study has several limitations. First, patient allocation was based on shared decision-making instead of randomization. Because the present study was not a randomized case–control trial, the objectivity of its findings could have been influenced by bias. Second, pressure-flow urodynamic assessment [30] was not conducted to evaluate the surgical outcomes of the patients in our study. To validate our findings, further research with a larger sample size is necessary. Third, it is imperative to categorize patients with urinary retention into subgroups, distinguishing between acute urinary retention and chronic urinary retention, since physiological implications for these two patient groups differ significantly. A detailed urodynamic examination is necessary to differentiate between them; regrettably, practical clinical considerations prevent us from conducting this in reality. Nevertheless, the present study is the first to investigate the use of endoscopic enucleation surgery in patients with UR. We believe that the present study holds considerable clinical relevance and generated reliable and valuable information for healthcare professionals.

## Conclusion

In the present study, both ThuLEP and B-TUEP demonstrated excellent therapeutic efficacy in treating BOO due to BPH. Notably, our results suggest that even though the UR group experienced greater changes in IPSS total, IPSS voiding, and PSA values relative to the non-UR group, the incidence of postoperative UR was not higher in the UR group relative to the non-UR group. Furthermore, our study suggests that the purported benefit of laser surgery in reducing blood loss is not prominent in patients with UR.

## Data Availability

The data used to support the findings of this study are available from the corresponding author upon request.
